# Recent advances in modified poly (lactic acid) as tissue engineering materials

**DOI:** 10.1186/s13036-023-00338-8

**Published:** 2023-03-20

**Authors:** Samanta Castañeda-Rodríguez, Maykel González-Torres, Rosa María Ribas-Aparicio, María Luisa Del Prado‑Audelo, Gerardo Leyva‑Gómez, Eda Sönmez Gürer, Javad Sharifi‑Rad

**Affiliations:** 1 Conacyt & Laboratorio de Biotecnología, Instituto Nacional de Rehabilitación, Ciudad de Mexico, Mexico; 2 Escuela Nacional de Ciencias Biológicas, Instituto Politécnico Nacional (IPN), Ciudad de Mexico, Mexico; 3 Escuela de Ingeniería y Ciencias, Tecnologico de Monterrey, Campus Ciudad de México, Mexico; 4 Departamento de Farmacia, Facultad de Química, Universidad Nacional Autónoma de México, Ciudad de Mexico, Mexico; 5 Faculty of Pharmacy, Department of Pharmacognosy, Sivas Cumhuriyet University, Sivas, Turkey; 6 Facultad de Medicina, Universidad del Azuay, Cuenca, Ecuador

**Keywords:** Biocomposite, Biotechnology, Fabrication, Nanotechnology, Poly (lactic acid), Tissue Engineering

## Abstract

As an emerging science, tissue engineering and regenerative medicine focus on developing materials to replace, restore or improve organs or tissues and enhancing the cellular capacity to proliferate, migrate and differentiate into different cell types and specific tissues. Renewable resources have been used to develop new materials, resulting in attempts to produce various environmentally friendly biomaterials. Poly (lactic acid) (PLA) is a biopolymer known to be biodegradable and it is produced from the fermentation of carbohydrates. PLA can be combined with other polymers to produce new biomaterials with suitable physicochemical properties for tissue engineering applications. Here, the advances in modified PLA as tissue engineering materials are discussed in light of its drawbacks, such as biological inertness, low cell adhesion, and low degradation rate, and the efforts conducted to address these challenges toward the design of new enhanced alternative biomaterials.

## Introduction

In the past few years, the regenerative medicine area has improved several achievements in looking forward to materials suitable for tissue engineering. A paramount concern of humanity is the implementation of techniques that favor the environment; such concerns have led us to develop materials with a lower ecological footprint. In a tangible field such as the environment in which we develop, there are factors to consider in reducing waste that, in turn, affect our ecological footprint on the planet. Besides, using toxic materials and biological waste for research is a latent problem [1]. To reduce the environmental impact and with the urgency of developing new materials, it has been sought to use polymers from natural and renewable sources extracted from bacteria, plants, or other organisms to implement innovative technologies such as tissue engineering with exponential growth in recent years.

Renewable resources have been sought to develop new materials, resulting in attempts to produce a wide variety of biomaterials that are friendly to the environment and low cost. However, the challenge in producing these new materials is that they must be biocompatible with organisms. In the field of tissue engineering, the biomaterials that are developed must be biocompatible and have a specific mechanical resistance to function as a support during the repair of damaged tissue. Using natural materials such as biopolymers can offer benefits for various applications, enhancing their properties, which are convenient for studying and treating diseases [2]. Poly (lactic acid) (PLA) is a biopolymer known to be eco-friendly, which has the characteristic of being a biodegradable polymer produced from the fermentation of carbohydrates. This characteristic allows it to be produced on a large scale and with reduced costs. Also, its production has low emissions of greenhouse gases [3]. This characteristic of biodegradability has been of great interest to tissue engineering due to its application for developing scaffolds and nanomaterials.

Moreover, the polymer industry kept growing until we were aware of these years, and PLA has been modified using various synthetic, semi-synthetic, or natural polymers to enhance its properties and thus design copolymers with new applications beneficial for biomedicine. Because of these advances, new applications have been updated, and new challenges for tissue engineering have been developed.

Recently, producing PLA and PLA-based derivatives for medical applications has received growing attention [4]. The use of emulsion, wet, blend, and coaxial electrospinning from PLA-based structures and their biomedical applications were reviewed over the last five years for the period up to the present day [5]. Similarly, a concise review focused on current kidney tissue engineering applications of PLA electrospun scaffolds [6]. Also, the review of the effect of PLA processing conditions on the physicochemical and biological material properties [7], the use of PLA-based microparticles for drug delivery [8], the use of PLA composites and blends for cutting-edge biotechnologies [9], PLA 3D printing [10], PLA membranes synthesis [11], and bioactive coatings of PLA for bone tissue engineering were highlighted and recently conducted [12]. The above reviews demonstrate that the topic of PLA derivatives designed to be used in biomedicine is fascinating, of much interest, and practical usefulness. However, the reviews carried out are particular for each topic, and to our knowledge, no review has collected the most recent and interesting works on the different PLA derivatives as well as the most suitable synthesis techniques for the use of this versatile biomaterial in tissue engineering, which is the main novelty and relevance of this work. Therefore, studying, highlighting and emphasizing the PLA intriguing history, the biosynthesis, the search of the different types of modifications and derivatives, as well as the recent advances of the most promising strategies for PLA use in tissue engineering is presented here.

### Inside the history of polymers: poly (lactic acid)

**Fig. 1:**
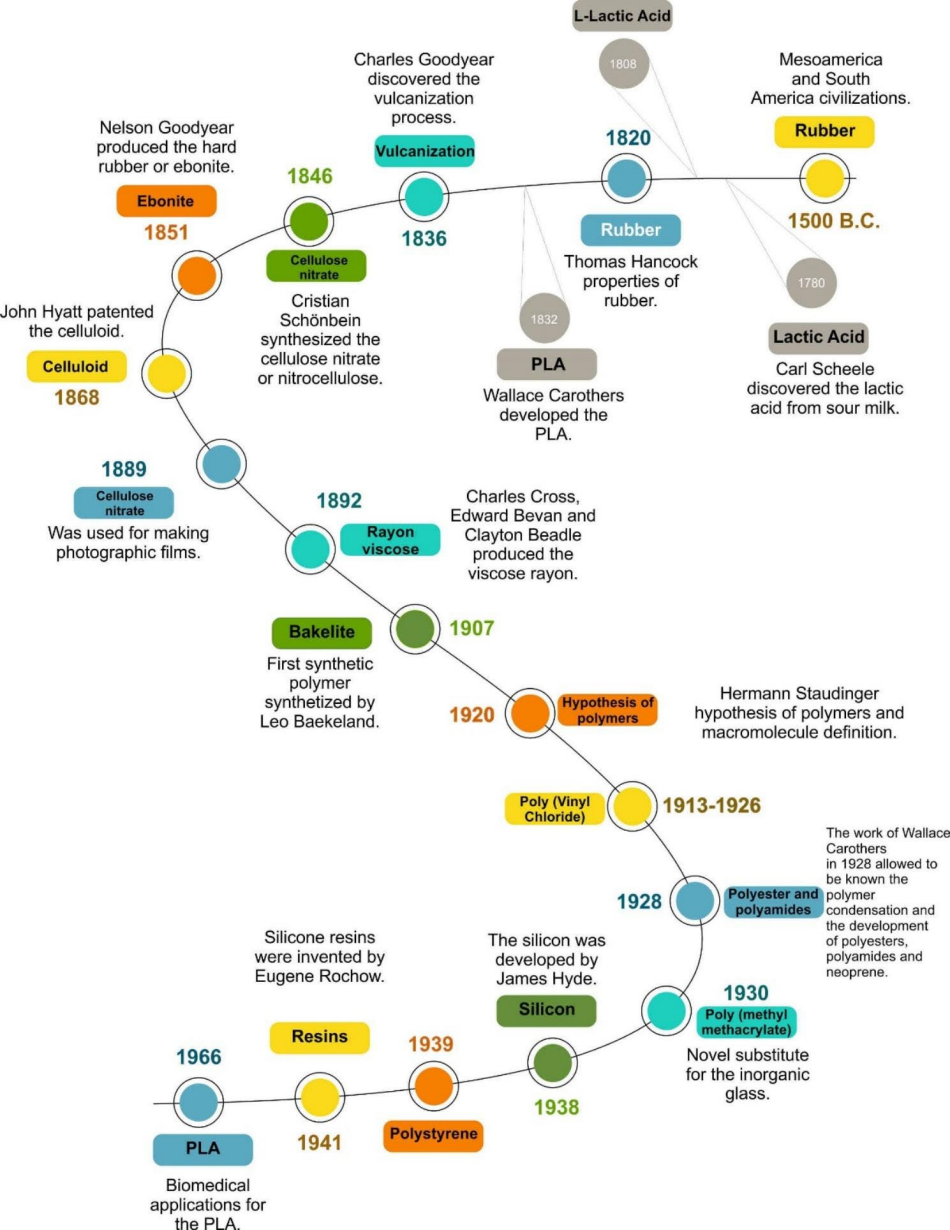
Inside the history of polymers

Lactic acid (LA) was discovered in 1780 by the Swedish chemist Carl Scheele from sour milk, and years later, Jöns Berzellius, in 1808, discovered the L-lactic acid, better known as L-Lactate, a molecule produced in muscles [13, 14]. According to Dorgan et al. [15], in 1832, Wallace Carothers developed the PLA when they tried to polymerizate and depolymerizate oligomeric lactides by polycondensation [16]. After that, in 1954, the PLA synthesis was improved to produce a high molecular weight, but it was costly. In 1966, Kulkarni et al. [17] established that PLA is a nontoxic, non-tissue-reactive, and slowly degrading compound that is possibly entirely metabolized through the respiratory system. Such discoveries have been the very beginning of the biomedical applications of PLA. As a result of the novel applications, the production of PLA increased to what we know today [Fig. 1].

### Lactic acid and poly (lactic acid)

**Fig. 2:**
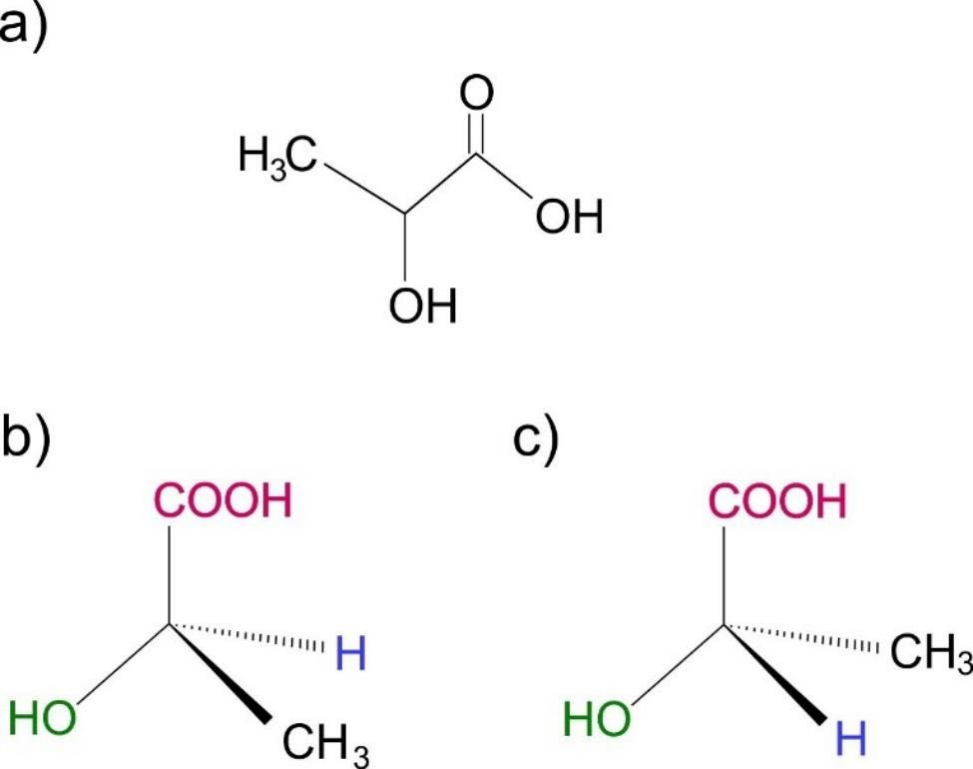
Structure of lactic acid. (a) The chemical structure of lactic acid (C3H6O3), the IUPAC name is 2 – Hydroxypropanoic acid. (b) Isomeric structure of lactic acid, (R) or L (+) Lactic acid. (c) Isomeric structure of lactic acid, (S) or D (-) Lactic acid

Poly (lactic acid) is an organic polymer derived from lactic acid, a chemical compound within organisms. As a result, lactic acid is one of the most important molecules in our bodies because it is a precursor in several metabolic pathways and is produced by animals, plants, and microorganisms. Besides, LA can be a good component of synthesizing other compounds because of the functional groups with which the lactic acid counts. The chemical structure of LA can be numbered in the hydroxyl and carbonyl groups. The LA, in its ionic form, is called lactate. Also, in the IUPAC nomenclature, the complete name is 2- hydroxypropanoic acid, a carboxylic acid with a hydroxy group in the α carbon, and the condensate formula is (CH_3_-CHOHCOOH) [18] [Fig. 2a]. Besides, lactic acid is a molecule with optical activity, which counts with a racemic mixture. The LA has three enantiomeric forms, which are L (+), D (-), and LD (+/-) [Fig. 2b and c]. In addition, the pure mixture of L-lactic acid and D-lactic acid has a high commercial value in the industry, and in fact, L-lactic acid is the chemical structure that is the monomer of poly (lactic acid) [19].

### Metabolic pathways and biosynthesis of the lactic acid

Lactic acid, from being a small organic molecule present in various organisms and participating in many biochemical processes, has the quality of not only being produced in the human body but also in microorganisms. In addition, it has given the possibility that lactic acid can be produced through biotechnological processes, which has allowed the advancement of the incorporation of biotechnologically modified strains or microorganisms for the development of new products [20–25].

Several microorganisms have been able to produce lactic acid and organic lactates, such as fungi [26, 27], cereal [28], yeast [29–31], cyanobacteria [32–35], and even algae [36], despite the great variety of microorganisms that produce LA, bacteria have been the most commonly used for the biotechnological production of lactic acid due to the ease and versatility of the handling and processing of bacteria. One of the most important genera of bacteria for producing lactic acid is the genera Lactobacillus. On the other hand, other bacteria produce LA, such as the Bifidobacterium genera, Bacillus Sporolactobacillus. Although these bacteria are not found within the group of lactic acid-producing genera, they include Enterococcus faecium, Lactococcus lactis, Pediococcus acidilactici, and Streptococcus thermophilus produce LA [37, 38]. Table 1 summarizes the most common lactic acid-producing bacteria and some of their characteristics.

**Fig. 3:**
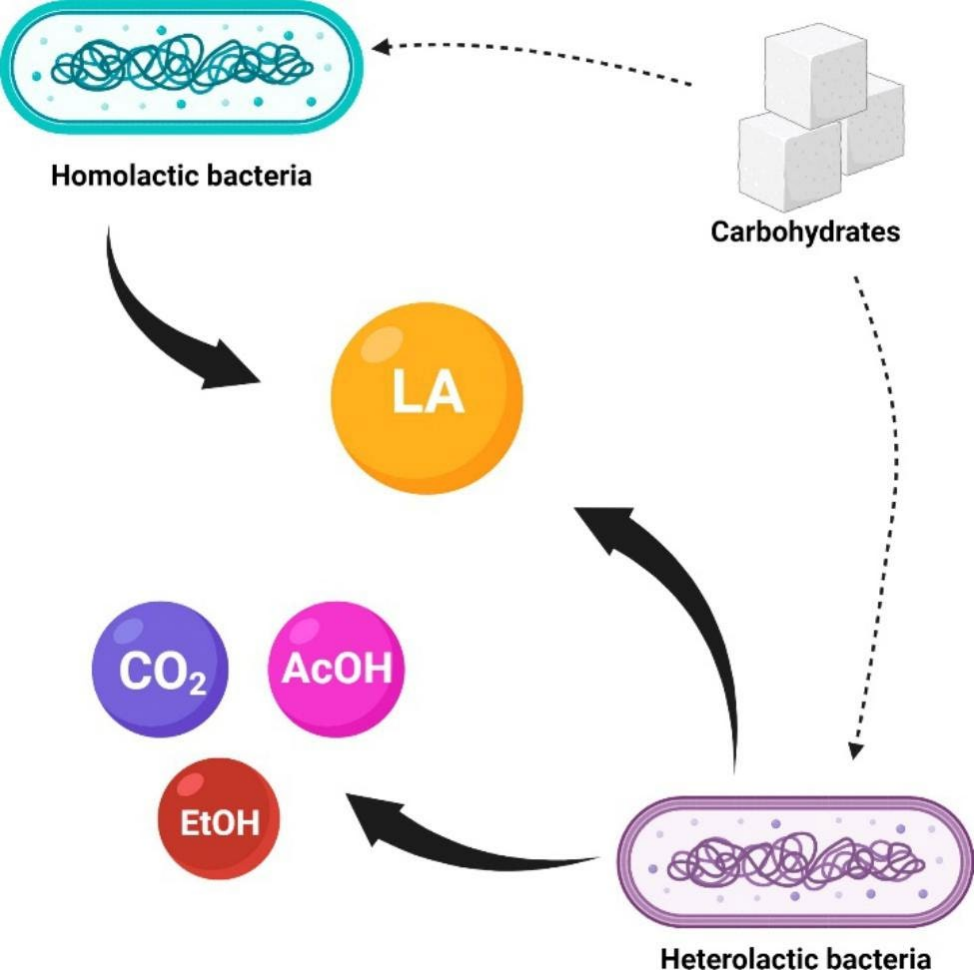
Products of fermentation of heterolactic and homolactic bacteria

According to Carr et al. [37], lactic acid bacteria can be classified into Homofermenters and Heterofermenters based on the type of production the bacteria can make. The homofermenters can produce LA by taking glucose as a product and transforming it by oxidation and fermentation. Moreover, the heterofermenters can produce other products instead the lactic acid; such products can be acetic acid, CO_2_, and ethanol produced by the fermentation of the glucose [Fig. 3]. It is worth mentioning that lactic acid bacteria are a broad group of gram-positive bacteria and are obligate fermentative [39]. The homofermentative bacteria can reduce hexose carbohydrates by the glycolysis pathway; the glycolysis breakdown the molecules of glucose or hexoses to turn them into pyruvate, and the primary production of lactic acid is generated by the enzymes lactate dehydrogenases (LDH) (EC 1.1.1.27).

**Table 1:** Summary of the most common lactic acid-producing bacteria and some of their characteristics

Genus	Shape	Metabolism	Microorganism	Growing conditions	Culture media	Refs.
Lactobacillus	Bacilli/pairs/chains	Homofermentative Heterofermentative	L. acidophilus L. delbrueckii L. brevis L. fermentis	T° opt.: 30–40 ˚C (2–53 ˚C) pH opt.: 5.5–6.2, tolerant < 4	Requires individually various complex nutritional requirements for peptides, amino acids, nucleotides, vitamins, and fermentable carbohydrates	[20, 22, 40, 41]
Lactococcus	Cocci/chains	Homofermentative	L. lactis spp. lactis L. lactis spp. cremoris	T° opt: 10 °C < 45 °C.	It may be selectively isolated on Elliker’s lactic agar, Arginine Tetrazolium Agar, or Alsan Medium. They usually grow in media containing 4% (w/v) NaCl	
Pediococcus	Cocci/tetrad	Homofermentative	P. acidilacti P. cellicola P. claussenii	Reduced atmospheric conditions	Pediococci grows on MRS media, and growth may be enhanced, as with the Leuconostocs.	[20, 22, 40, 42]
Leuconostoc	Pairs/chains	Heterofermentative	L. mesenteroides L. cremoris L. oenos	Alkaline environment, pH opt: ≥ 4.5.	Although MRS agar is suitable for Leuconostocs, Yeast Glucose Phosphate Peptone Broth is recommended.	

Also, the LDH enzyme family can exist in an L or D stereospecific form [43]. In addition, those enzymes are oxidoreductases, and the LDHs can be classified into two groups, the NAD-dependent LDH, and the NAD-independent LDH. LDH NAD-dependent enzymes catalyze the reaction. In other words, they depend on NADH’s oxidation to NAD+. The L-LDH NAD-dependent catalyzes a redox reaction in which the pyruvate is reduced into L-lactate or L-lactic acid; those enzymes can generate the reaction reversibly or irreversibly. In this, the final product of glycolysis is lactate. According to Garvie [44], the LDH enzymes can differ between species existing in different stereoisomer forms for the L or D forms, dependent or non-dependent forms.

**Fig. 4:**
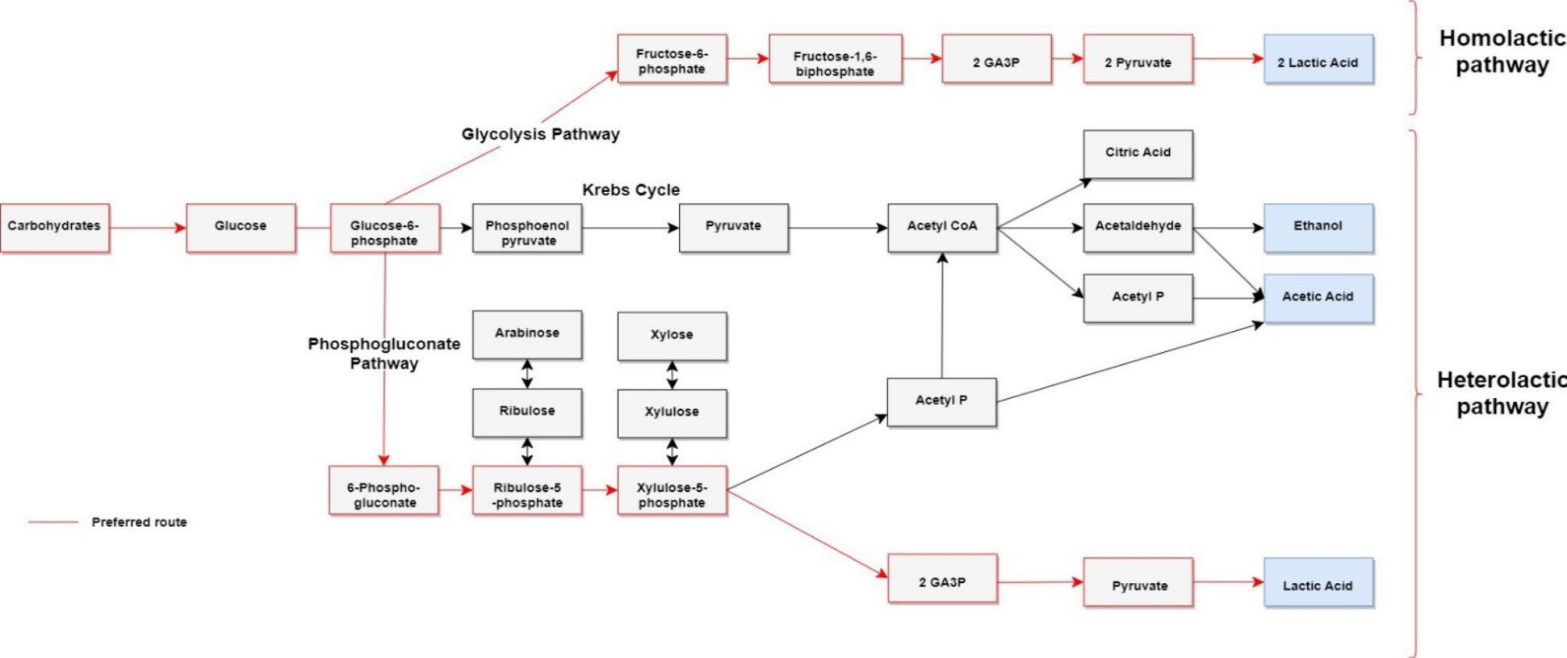
Summarized homolactic and heterolactic pathways of lactic acid bacteria

In contrast with the homolactic fermentative bacteria, under anaerobic conditions, the heterolactic fermentative bacteria use the phosphoglucanate path, commonly known as the phosphoketolase pathway, to earn LA, acetic acid, ethanol, and CO_2_ [45–47]. The phosphoglucanate pathway transforms the glucose or hexoses into pentoses through the enzyme phosphoketolase (EC 4.1.2.22) [48]. The enzyme catalyzes a reaction producing glyceraldehyde 3–phosphate (G3P) and acetyl phosphate [Fig. 4]. If a pentose enters, the path does not yield CO_2_ [49, 50]. The phosphoketolases (Pkts) catalyze an irreversible reaction and play together with other enzymes, like acetate kinase and phosphotransacetylases, to produce acetate, ATP, and acetyl Co-A [51]. Also, G3P is oxidated in pyruvate and lactic acid. On the other hand, at the end of the reaction, the acetyl phosphate turns into ethanol [22, 52, 53]. In humans, as in homofermentative bacteria, the lactate is produced by glycolysis using glucose to transform it into pyruvate and, by the action of the LDH, is converted into lactate by a reversible reaction with NADH as a coenzyme. In the human body, lactate is a waste product of anaerobic metabolisms and is used in gluconeogenesis to develop energy by oxidation in metabolic pathways like the Krebs cycle and Cory cycle; as a result, the skeletal muscle is the first consumer of lactate in the organism [54].

Also, the accumulation of lactate in the organisms, to be more specific, the accumulation of lactate in the blood, can bring consequences to the health and the generation of lactic acidosis [55–57].

### Polymerization of poly (lactic acid)

Two different methods can prepare the polymerization of PLA. The first method is polycondensation, and according to Garlotta [58], the polymerization of lactic acid results in a low molecular weight polymer, brittle, glassy polymer, and is unusable unless mixed with other substances to increase its molecular weight.

**Fig. 5:**
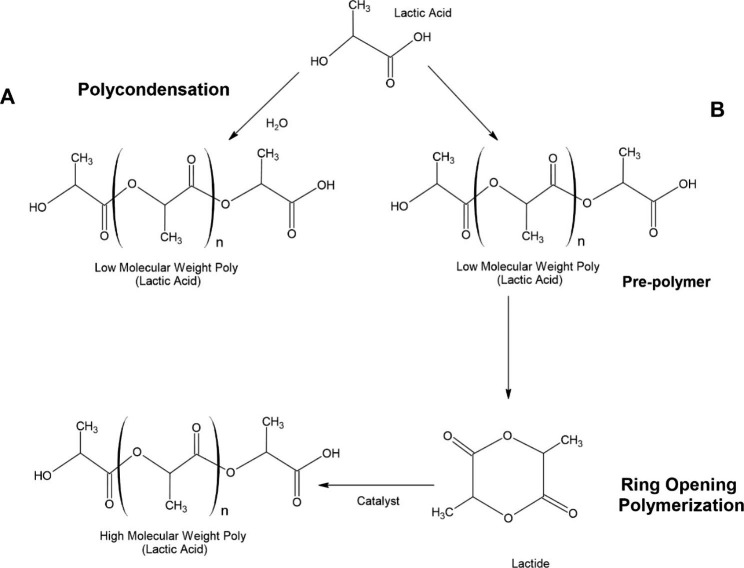
Synthesis of poly (lactic acid). (A) The polymerization of lactic acid by polycondensation results in a low molecular weight polymer, water is removed from the solution under elevated temperature and high vacuum conditions. (B) The generation of Lactide a cyclical intermediary, allows the ring-open polymerization (ROP) where under specific conditions (heat and accurate solvent) a high molecular weight polymer is obtained, with ROP a pure polymer and a higher yield of the reaction is obtained

In addition, the molecular weight of this polymer is low due to certain factors such as viscosity, impurities, water in the molecule, and the low concentration of reactive end-groups. In the polycondensation method, as its name indicates, water is removed from the solution by condensation using the solvent under elevated temperature and high vacuum conditions; a polycondensation reaction in regular terms is conducted in bulk via distillation by condensation of water. Likewise, the transesterification process is accelerated if a reaction catalyst is added. This lactic acid polymerization method allows the synthesis of PLA oligomers. Although the average molecular weight of the polymer is low compared to other methods mentioned, it allows the addition of other compounds to increase the molecular weight of the polymer [59]. The second method consists of generating a new molecule whose function is to be a cyclical intermediary; this intermediary is called lactide. The polymerization method is by ring-open polymerization (ROP) where under specific conditions, such as heat and without the need for a solvent, a high molecular weight polymer is obtained; this method allows the polymerization process to be conducted, which favors us in obtaining a pure polymer, guaranteeing a higher yield of the reaction. According to Metha et al. [60], a catalyst frequently utilized to conduct this reaction is stannous octoate from zinc metal. In addition, the choice of catalyst, initiator, and co-initiator for this ROP reaction affects the properties of the PLA polymer. A disadvantage of this type of polymerization is that conducting this process is unfavorable due to the high costs of polymerization. However, the development of innovative technologies such as membrane design, ultrafiltration, chromatography, and electrodialysis, among others, has allowed purification costs to decrease the PLA and therefore improve the processes, making it possible to obtain more efficient products. Figure 5 illustrates the types of polymerizations of polylactic acid [18] [Fig. 5].

### Characteristics and properties of PLA

PLA has a wide range of characteristics and properties. Hagen [25] describes that PLA is a transparent glass that is opaque when crystallized. In addition, Table 2 summarizes some characteristics and properties of PLA.

**Table 2:** Properties of poly (lactic acid)

Properties	Value	Refs.
Melting temperature	Tm = 230 < 240 °C	[61, 62]
Equilibrium Tm	Tm = 165–279 °C	[61–63]
Glass transition temperature (Tg)	Tg = 65–72 °C	[59, 62, 63]
Crystal form	Trigonal	[61, 64]
Melting enthalpy (ΔHm)	142–155 J/g	[62, 65]
Density	1.21–1.34 g/cm^3^	[66]
Tensile strength	80 Mpa	[59, 62, 65]
Young’s modulus	8.6 GPa	[67]
Elongation	30%	[59, 67]
Hydrolytic degradation	PLA fibers 40% ~ 25 days	[63]
Viscosity	At room 1.258 g/cm^3^ temperature	[66]
Permeability	Water 2954 ± 120 CO_2_ 32 ± 7 O_2_ 6 ± 0	[68]
Rockwell Hardness	~ 70–90	[67]

### Insight into PLA degradation mechanism

PLA can be subjected to enzymatic, hydrolytic, microbial, and ultra-violet degradation (photodegradation). These processes are essential to determine the long-term impact of PLA-based materials. From a biomedical point of view, PLA degradation produces a decrease in crystallinity percentage and molecular weight and generates smaller devices. The PLA’s low degradation rate is not conducive to biomedical applications, especially bone tissue engineering. Practical strategies to address this drawback are grafting, copolymerization, compounding, and blending with other substances. It is very challenging to find a balance between degradation rates, mechanical strength, porosity, conformation, degree of crystallinity, shape, chemical cues, biodegradability, and biological activity to develop devices that ensure tissue’s safe growth and sustain the biomaterial stability to perform the function for which it was implanted [69–71].

### Modifications of PLA as a tissue engineering material

Decades ago, we could barely imagine the possibility of developing biomaterials using nanotechnology to improve humanity’s quality of life. Today, biomaterials are widely used in endless applications, ranging from the food industry, textiles, and the medical and pharmacological industries, among others. A lot has been said regarding the applications of biomaterials in various areas of study or innovation. However, the field of biomaterials still has much to be explored. One of the multiple applications of biomaterials is the use of biopolymers as innovation materials in tissue engineering, for which the development of biomaterials focused on the treatment of diseases has allowed the enhancement or improvement of cellular activity, serving as scaffolds for cell support or transport of pharmacological molecules that improve cell activity or viability allowing new treatments.

Additionally, an essential aspect of the development of biomaterials is the use of characteristics and functionalities selected from biological systems that provide us with essential information that can be utilized to develop new biomaterials that imitate the mentioned biological systems. For example, modified biopolymers mimic various aspects of the human body; according to Green et al. [72], hydrogels allow the creation of structures similar to the human body as scaffolds for in vivo tissue repair and for the cultivation of Stem cells. In addition to the above, the use of biopolymers in tissue engineering has characteristics of great interest, among which we can count on biocompatibility, bioactivity, non-toxicity, biodegradation, adaptable mechanical properties, and a biopolymer synthesis process of great convenience; all of these focused on the improvement of biological structures that mimic the body despite the use of synthetic polymers that although this might seem to generate incompatibility problems. The use of these natural or synthetic biopolymers allows adaptability to generate ideal materials that allow the functionality of the tissue, in addition to the fact that the area of tissue engineering and regenerative medicine emphasizes the use of these novel techniques for the essential goals of allowing the repair of damaged tissues or organs and that they continue to have origin from biofunctionality [73–78].

**Fig. 6:**
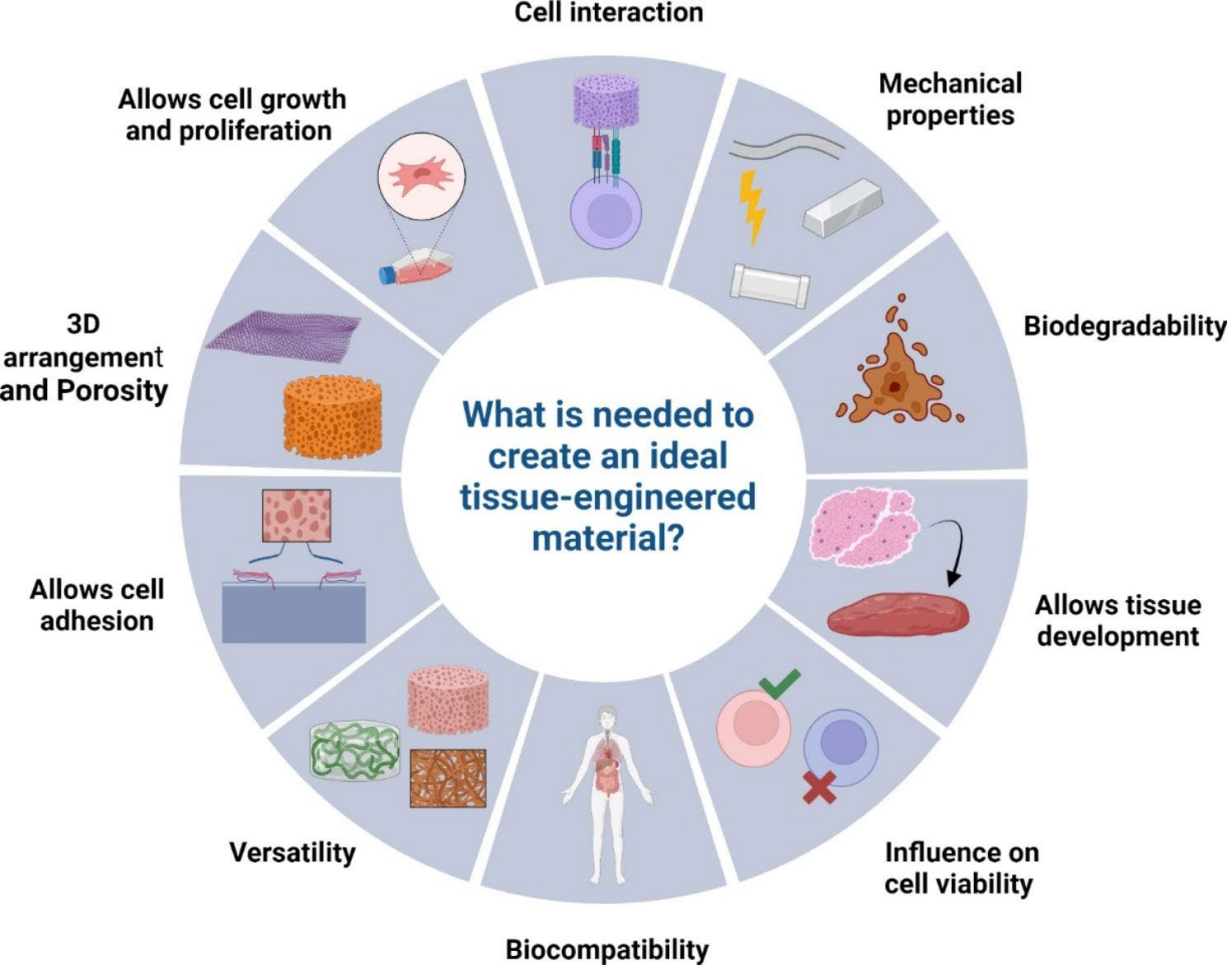
Ideal tissue engineering material. Diagram of some relevant characteristics of an ideal tissue-engineered material for biomedical applications

Considering the broad characteristics of materials, one question remains: What is needed to create an ideal tissue-engineered material? The cells’ interactions with the materials must be considered to answer this question and how this interaction works to develop a tissue. One of the most popular characteristics is that the material has to be biocompatible with the cells and with the human body, which is essential to avoid immunogenic responses like allergies or rejection of the material from the body like a foreign body response, also with the compatibility, the toxicity is another relevant characteristic to have an ideal material. The polymer has to be safe for the organism and probe that the materials are not carcinogenic or have a potential risk of causing an illness. Also, biodegradability can be important too. A material that allows the regeneration of the tissue while it is disappearing can bring support to the cells and helps to promote the proliferation, migration, and cell growth in the scaffold. Cell interaction with the material is also needed to understand how the type of material and porosity influence the viability of the cells, or if the cells tend to create aggregates, it benefits the tissue development and how the cell adhesion works with a predeterminate type of material. The 3D arrangement of the scaffolds influences cell differentiation and the phenotype of the cells. Another last but not least important characteristic is the versatility of the polymer because it is advantageous to have materials that can be transformed into fibers, vesicles, or hydrogels to match biomedical applications [79–81] [Fig. 6].

As an emerging science, tissue engineering and regenerative medicine focus on developing materials that replace, restore or improve tissues or organs and enhance the cellular capacity to proliferate, migrate and differentiate into different cell types and specific tissues, respectively [82–84]. Therefore, the importance of these areas of the study lies in current therapies for various diseases ranging from the regeneration of skin, blood vessels, cartilage, heart tissue, bone, brain disorders, and even further in the urinary tract or gastrointestinal tract [85–91].

### PLA as a tissue engineering material

Poly (lactic acid) has proven to be a biopolymer with great functionality in the biomedical area; considering the characteristics and properties of PLA that offer a large set of benefits and that combined can generate medical devices, we can highlight that within the properties of the biopolymer, the most important are biocompatibility, non-toxicity, and biodegradability [92].

Now, the central question is why the PLA needs to be modified. The copolymerization of PLA or the mixture of PLA with other polymers allows PLA to improve its properties and biological functions. According to Cheng et al. [93], the factors that influence the properties of polymers are the chemical components, the composition, and the morphological structure, among others. The authors also mention that they can improve the properties of both polymers. For example, the union between PLA and poly (glycolic acid) (PGA) results in a polymer with better properties such as low crystallinity and melting temperature (Tm), an example of an improved property is the copolymer poly (lactic glycolic acid) (PLGA), the concentration of the monomers can adjust the degradation of this biopolymer. Therefore, the modification of PLA allows that when grafting or making a mixture of other polymers with PLA, aspects of interest are modified according to the types of different polymers grafted to the base chain of PLA. In addition, a highly relevant benefit of PLA blends with other polymers is that when grafting or blending a new copolymer, it can be focused on a specific application that generates new research areas. Another benefit of PLA copolymerization is that the properties and characteristics, such as hydrophobicity of PLA, can be masked by other polymers and that mixture improves the capacity of PLA for more excellent compatibility [94]. However, it must be considered that some biopolymers are incompatible with PLA due to their hydrophobicity since, being an aliphatic compound, it tends to differ from hydrophilic compounds, leading to low performance and inadequate performance response of the copolymer properties [95]. Likewise, chemical modifications are made to improve the crosslinking of the polymer, and this characteristic is relevant for synthesizing hydrogels since these modifications allow the crosslinking between polymers to be obtained with greater efficiency and ease [96].

### Overview of some advances in PLA modifications: an insight into tissue engineering

According to the benefits of PLA copolymerization and its various properties, new biomaterials based on biopolymers have emerged due to the focus on improving treatments for various diseases.

### PLA/Hydroxyapatite (HA)

One of the rising biomaterials developed is PLA modified with HA. HA has turned out to be a ceramic material with enormous potential for tissue engineering. It focuses on bone since the bone tissue in the crystalline phase is made up of HA in its natural state. In addition, ceramic materials such as HA have a significant porosity that allows this material to blend with bone tissue and bring oxygen. The biological importance of using HA in scaffolds is the natural presence in the bones. Therefore, HA increases the Ca^2+^ of the cell, thus allowing the proliferation of osteoblasts and promoting cell growth [97, 98].

On the other hand, PLA can be 3D printed for the design and synthesis of scaffolds that allow application in bone tissue engineering. Despite its outstanding biocompatibility, PLA has the disadvantage of not having suitable cell adhesion, which sometimes leads to the material being discarded for cell growth. However, this disadvantage can be overcome because when mixing HA, which is known to allow excellent cell adhesion and, therefore, cell proliferation, it allows cell adhesion by exerting electrostatic interactions with cell protein receptors; in this way, the capacities of the material would be balanced to be a candidate for the treatment of fractures or injuries where the bone can no longer regenerate itself [99, 100]. An exciting aspect worth mentioning is that for bone tissue engineering purposes, modifications to the surface of the scaffold can be made by adding molecules that improve cell adhesion.

One of the most versatile techniques that allow obtaining nanofibers with great ease and performance is electrospinning, which allows fibers to be created from a mixture of biopolymers in the order of nanometers to micrometers; this is attractive for the design of scaffolds. Another technique is air spray spinning, which covers areas with the compound of interest. This technique can help design and synthesize mats used as a scaffold for cell migration [101–104].

Currently, PLA polymeric fibers have been developed using the rotary jet spinning (RJS) method, which allows generating effective wound dressing for skin tissue engineering. RJS provides several advantages, such as wound healing acceleration and improved cell proliferation, migration, and adhesion. Also, the resulting fibers offer adequate porosity and surface area while mimicking or imitating the extracellular matrix’s structure well [105]. Recently, the RJS method permitted the production of antibacterial poly(ε-caprolactone)/Poly (lactic acid) fibers loaded with Vancomycin, which showed potential for use as a dressing intended for wound repair [106]. Furthermore, other research on newly developed PLA fibers combined the polymer with curcumin to produce a membrane by RJS for wound healing applications. The authors demonstrated that PLA / curcumin membranes are cytocompatible with mouse embryonic fibroblasts [107]. It is worth mentioning the work highlighting the nature, wettability, morphology, and thermal properties of non-woven PLA produced from the RJS process. The quality and hydrophobic nature of the fibers were exhaustively assessed [108]. Besides, an exciting work reported the production via RJS of highly aligned and controlled nanofibers. The study described the dimensionless parameters used to prepare and scale up reliably nanofibers of several polymers, including PLA [109]. Lastly, it is of note a work employing centrifugal spinning for producing PLA / gelatin ultrafine fibers that were suitable for skin tissue engineering when loaded with ciprofloxacin [110]. Conclusively, the RJS method offers unique advantages and versatility to produce diverse types of fibers and scaffolds with controlled properties beneficial for tissue engineering.

On the other hand, carrier scaffolds of PLA combined with HA and poly (ethylene glycol) (PEG) have also been used for the transport of recombinant proteins that induce bone tissue formation, which allows an improvement in cell proliferation to repair the damaged tissue. Also, the use of erythropoietin as a precursor for proliferation and pleiotropic effects related to bone tissue [101, 111]. Even the use of PLA/HA coated with polypyrrole (PPy) has been reported, favoring cell proliferation due to its conductive properties [112]. Likewise, the PLA/HA copolymer has been used as a scaffold for regenerating dental pulp tissue that can help with treatments ranging from dental trauma to congenital disorders [113].

Another approach of the PLA/HA copolymer has been found for cartilage repair, where the biomaterial is used as a scaffold to carry recombinant proteins that will be precursors of cell proliferation of stem cells to differentiate into cartilage [114].

### PLA/Poly (glycolic acid) (PGA)

The PLA/PGA copolymer has turned out to be a versatile biomaterial that has focused on the engineering of musculoskeletal tissues such as bone, menisci, or cartilage because when trauma occurs, disease or congenital abnormalities exist. It is difficult to recover the tissue when it is damaged or lost; thus, using this material as a scaffold allows the replacement or regeneration of chondrocyte cells or osteoblasts. Likewise, it has been reported that PGA is an excellent biomaterial for cell growth, in addition to the fact that the mixture with PLA gives it greater effectiveness concerning cell growth and adhesion [115–119]. One of the characteristics that stand out and is convenient for the development of scaffolds for various tissues is the appropriate biocompatibility that this copolymer has and in addition to the fact that it can be designed in different structures such as scaffolds or nanocomposites for various biomedical applications such as, in the regeneration of trachea, spinal cord, and the brain. The latter case is of great relevance in the current biomedical area since being able to develop biomaterials that allow the regeneration of damaged brain tissues is a premise for neurodegenerative diseases using scaffolds with Schwann cells and neural Stem cells. Unlike other applications for this copolymer, the mechanical strength is not essential, but the biodegradation rate is determined by the polymer’s crystallinity and molecular weight. According to Agrawal et al. [120], the PLA/PGA copolymer degradation rate depends on the exact rate of PLA and PGA monomer contained in the polymer, and this biodegradation is conducted by breaking ester bonds hydrolytically. Likewise, the modifications of the topology of the copolymer open an opportunity to obtain more excellent cell adhesion and, thus, better cell proliferation and differentiation [121].

On the other hand, another relevant application is the regeneration of bone tissue for facial reconstruction, a common injury caused by trauma [122]. Another function of the scaffolds of this copolymer is focused on the regeneration of cardiac tissue, which is a well-known heart disease and is a latent risk today. Therefore, the focus on cardiac tissue regeneration is vital for current research. Tissue engineering allows the development of biografts with skeletal myoblast and endothelial cells, among others, to produce a scaffold that carries the cells allowing correct oxygenation, adhesion, cell proliferation, and angiogenesis, as in the scaffolds for neuronal tissue. The rate of degradation in cardiac tissues is essential and has adequate in vivo prevalence to migrate the cells, and the proliferation process occurs adequately [123, 124]. A last point of interest is the use of PLA/PGA nanofiber scaffolds with drugs for their administration, and an example is the use of PLA/PGA/Ibuprofen nanofibers for administration in chronic wounds and to help relieve pain caused by these diseases. These scaffolds adhere to cells and release drug-producing benefits such as pain reduction and relief of wound inflammation [125].

### PLA/Poly(butylene-adipate-Co-terephthalate) (PBAT)

There is a wide range of applications for the mixture of PLA and PBAT polymers, mainly using electrospinning techniques for the generation of nanofibers of these compounds, in which different modifications can be made to parameters such as the types of solvents, mixing ratio of binary solvents, polymer blend concentration, polymer blend ratio, among others [126, 127]. This combination of compounds has also been used in other areas of study, for example, for the production of films, resulting in better tensile strength and significantly improving the joint use of both compounds than in isolation [128]. On the other hand, these polymers have been used to manufacture biomembranes and as materials in food packaging, resulting in a high degree of versatility and a wide range of applications [129].

Other investigations have shown that both compounds have excellent biocompatibility, resulting in promising materials used in bone tissue engineering [130]. This mixture has also favored the proliferation of fibroblasts since it has accessible mechanical properties, high porosity, well-connected microporous structures, excellent water permeability, and good biocompatibility to support the formation of new tissues, allowing these cells to maintain their phenotypic shape. As the PBAT content increased, the mean diameter of the PLA/PBAT scaffolds decreased while the mechanical properties improved [131, 132].

### PLA/PEG

Even though there are many ways of designing biomaterials, hydrogels allow a favorable environment to be cell-carrying scaffolds. PEG has the characteristic of improving the biocompatibility, hydrophilicity, ductility, and flexibility of the copolymers. However, PLA is a polymer with low hydrophilicity; in addition to being molded in different structures, they have the characteristic of having significant porosity, which is of great help for cell proliferation and oxygenation, and this, in turn, is of great interest for bone tissue engineering because it forms a versatile matrix to serve as a carrier scaffold for cells or recombinant proteins [133–135]. Taking advantage of PEG’s good miscibility with organic solvents and its biodegradation by hydrolysis, and that PEG surfaces can be modified, they can be used to develop micelles that can be drug vehicles or function as materials with antibacterial activity. According to Tessmar et al.[136], an attractive property of PEG is that because it is an uncharged molecule, it tends to form highly hydrated polymer coils on the copolymer surfaces, and most importantly, it can repel proteins. This property is used because, with modifications, it is possible to obtain micelles or other biomaterials with specific interactions resulting from peptide sequences grafted to the copolymer.

In addition, when PLA/PEG are copolymerized, they have the characteristic of improving PLA degradation properties, biocompatibility, non-toxicity, and good solubility, which allows scaffolds to be designed with good porosity, resistance, and degradation. Applying the copolymer to wound healing is the implementation of platelet growth factors to induce cell proliferation and thus have a better regenerative process [137–139].

Regarding the application of bone tissue engineering, the porosity property of PLA/PEG material has allowed the development of three-dimensionally printed scaffolds. Also, the use of techniques such as electrospinning that allows the creation of nanofibers for the design of mats that are of great support for the synthesis of scaffolds where cells can have excellent adhesion, proliferation, migration, and nutrition of Stem cells since it has been reported that Stem cells have had a more significant differentiation in their potential because they favor the expression of osteogenic cell markers [140–142].

### PLA/Lignin

Cellulose is not only one of the most critical polysaccharides, but also lignin is one of the most abundant polysaccharides all over the world. Because of the above, lignin has excellent potential to be used as a polymer in multiple biomedical applications since this polysaccharide has properties of great interest, such as antimicrobial, antioxidant, anti-ultra-violet (UV), biocompatibility, and non-toxicity properties [143].

Within the various applications for the PLA/Lignin copolymer, the use of scaffolds made from nanofibers by electrospinning focused on cartilage and bone tissue engineering is one of the leading applications today [144–146]. In addition to the fact that the PLA/Lignin copolymer has the characteristic of enhancing the properties of mechanical, thermal, and UV resistance and provides it with the essential characteristic of improving resistance to oxidative stress. The contributions of lignin enhance the properties and add new ones with which PLA alone does not count. This fact makes the copolymer attractive for the development of biomaterials, such as the development of PLA/Lignin films through physical methods like the mixture of these two polymers for various applications in the biomedical area with the use of Stem cells [147].

A novel application of the PLA/Lignin copolymer is the use of PGA to make a nanoparticle that has drug delivery functionality with a specialized focus on those therapies that are difficult to administer or have a beneficial result for patients; an example is the use of these nanoparticles to improve drug delivery efficiency for patients with triple-negative breast cancer. In this example, lignin improves the drug delivery system, and the copolymer improves cell non-toxicity and the biocompatibility of the nanoparticle, in addition to the fact that thanks to its compact size, it makes it a useful resource for drug delivery by nanoparticles [148]. In addition to the fact that a property of lignin that is of foremost importance is that it can form porous materials, and this is of great interest due to its multiple applications in tissue engineering as a common element for the creation of scaffolds for the regeneration of damaged tissue [149].

### PLA/Poly(pyrrole) (PPy)

One of the most promising materials due to its high conductive properties is PPy, an inorganic polymer. Polypyrrole has been considered one of the most useful in studying neuronal regeneration. Due to this, tissue engineering focused on neuronal tissues has used PPy as a polymer in combination with various polymers to develop scaffolds. One of these exciting materials is PLA. Together with PPy, they make up one of the most valuable materials because PLA is a highly biocompatible polymer with the organism and has high degradation capacities in conjunction with PP and PLA, which gives it these characteristics, enhancing biocompatibility. Something of considerable interest for neural tissue engineering is the ability of the material to electrically stimulate the proliferation, adhesion, and cell growth of damaged neuronal tissue. However, it is also of great interest for other tissues with electroactive potentials, such as the heart [150].

Among the applications of the copolymer is the creation of nanofibers by electrospinning of PLA/PPy, according to Tian et al. [151] report that the copolymer nanofibers were found to have better adhesion, viability, and cell proliferation. Then, the development of PLA/PPy nanofiber scaffolds to support bone marrow stem cells with the premise that cell regeneration is induced in spinal cord injuries. It is worth mentioning that these studies have been conducted in vivo in Winstar rats [152]. Another application has been made with the development of conductive fibers with conductive centers of PLA/PPy. Surface modifications were made to these fibers by adding proteins to make a bioactive scaffold that allows better adhesion and biocompatibility while preserving electroactive properties. These properties are of great interest for repairing damaged tissues, in addition to the fact that these scaffolds can be electrically stimulated, which allows the support of cell adhesion [153, 154].

### PLA/Chitosan (CHI)

Chitosan is a natural polymer made from renewable resources obtained from waste from the fishing industry and the shell of mollusks. It is a biocompatible, biodegradable material with antibacterial activity and allows wound healing [155, 156]. Additionally, chitosan and its derivatives are promising candidates to serve as support material in tissue engineering applications because of their characteristics of porous structure, gel-forming properties, ease of chemical modification, and high affinity with macromolecules in vivo, among others [157].

In tissue engineering, there has been a more remarkable boom in the study of the regeneration of damaged tissues as an alternative to autografts, where the mixture of PLA and chitosan stands out [158]. Nowadays, a great variety of natural and biocompatible compounds have been used for the elaboration of scaffolds with applications in regenerative medicine, chitosan being the material of choice since scaffolds present antibacterial and proangiogenic activity and mimic the extracellular matrix [156, 159]. In addition, one of the main limitations of PLA is its low cell affinity because it has poor cell recognition sites and low hydrophilicity; for which chitosan has allowed us to overcome these limitations of the scaffolds of PLA since it has minimal reaction to foreign bodies and good hydrophilicity, has been used as a surface modification material to improve cell attachment and proliferation in scaffolds [160]. Another advantage of the mixture of PLA and chitosan polymers is that it reduces acid by-products which can cause inflammatory reactions in the tissues and generate clinical failures; the above were present in the scaffolds that were only composed of PLA [161]. One of the most used techniques for producing PLA and chitosan nanofibers is electrospinning, and it is intended that these spun nanofibers be used in the native extracellular matrix for tissue engineering [162].

An example of the use of these scaffolds made by electrospun emulsion is in the regeneration of bone tissue, which turned out to be compatible with cells and biodegradable for periodontal bone regeneration by regulating their mechanical and biological properties; chitosan also promoted cell adhesion and osteogenic differentiation of bone marrow stem cells (BMSCs) [163]. Another example of its use in the regeneration of bone tissue is in preparing a tunable biomimetic matrix composed of chitosan, which promotes osteoconduction, and positively affects the behavior of osteoblasts [164]. In the same way, multiple applications of scaffolds composed of PLA and chitosan fibers have been found for cardiac tissue engineering and to accelerate myocardial regeneration since, in a particular proportion, they support the viability of cardiomyocytes, cause cell elongation and enhance the production of sarcomeric α-actinin and troponin I. On the other hand, PLA and chitosan nanofibers have also been used for the treatment of cuteness injuries caused by burns. These three-dimensional scaffolds were made from electrospinning techniques and are not toxic to skin cells, and can mimic the extracellular matrix, mainly composed of nanofibrous proteins[165].

### PLA / Poly (Caprolactone) (PCL)

One of the mixtures with the most significant potential in tissue engineering is the one conducted with PLA and PCL. These polymeric compounds have allowed the realization of scaffolds with multiple applications in regenerative medicine; their main advantages are their high purity, adequate processing, and excellent mechanical properties. Likewise, it should be noted that they are biodegradable materials whose degradation products can be reabsorbed.

Lactic acid presents various physiological and metabolic pathways from being a small organic molecule. The addition of these two compounds enhances the biomechanical performance of the constructions, and some studies have shown improvements in their mechanical and biological properties [166, 167].

Among the primary uses of these materials is the construction of bone scaffolds using an indirect 3D printing approach, in which the cells of interest became viable and proliferated, as well as increased biocompatibility and osteoinduction properties [168]. Another application of this mixture is in blood vessel tissue engineering, where scaffolds have been developed that mimic their architecture through sequential electrospinning technologies, resulting in excellent candidate scaffolds for this area. In the same way, its enormous potential for application in the field of vascular patches has been demonstrated [169, 170]. Some research indicates that the expression levels of elastin, angiopoietin, laminin-4α and − 5α increased in PCL and PLA nanofibers without any exogenous factor, in addition to the fact that they are significantly less hydrophobic and have less resistance to traction [171]. Given the above, it should be noted that the high biocompatibility between both compounds and their physical properties make them a suitable material for the replacement of blood vessels, which in the future would allow the possibility of functionalizing that material with a variety of molecules and modulate inflammatory and coagulative responses. Then, suitable devices would be obtained to replace native vessels [172, 173]. Other examples of composite grafts exhibited significant improvements in mechanical characteristics compared to single-material devices, particularly in compression and torsional strength, which are common problems with single-polymer vascular grafts compared to composite vascular grafts[174].

**Fig. 7:**
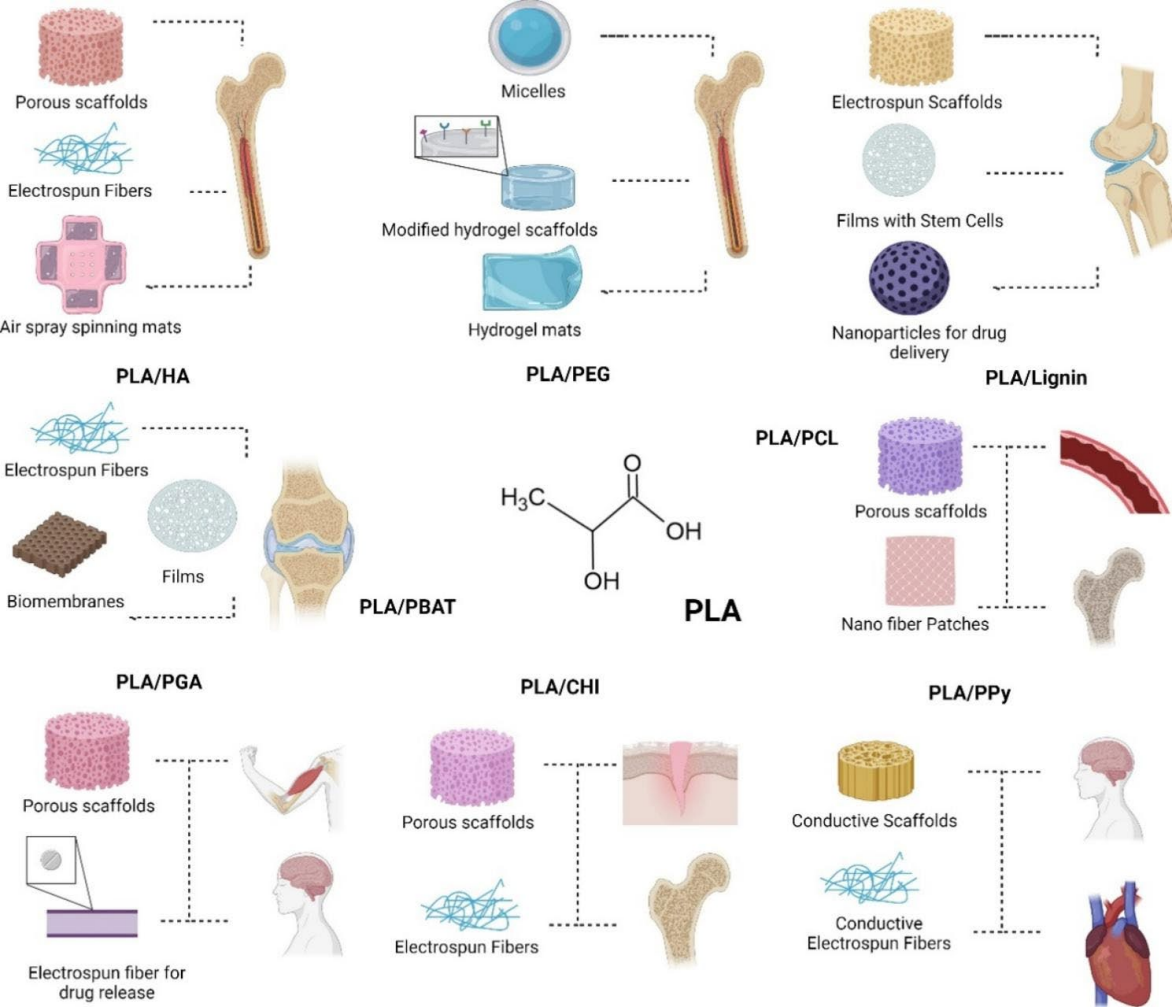
Poly (lactic acid) modifications and applications

In addition to its use in the regeneration of vascular tissue, the mixture of PLA and PCL polymers has made it possible to develop viable nerve tissue substitutes by combining scaffolds with transplanted cells and growth factors. Inkjet technology is attractive for manufacturing these scaffolds due to the incorporation of non-contact approaches that allow precise volumes of material to be deposited with high speed and precision at destination sites [175]. Figure 7 summarizes the previous section in a graph showing the most common PLA blends and their applications. Also, Table 3 summarizes the PLA combinations mentioned above, highlighting advantages and some relevant properties.

**Table 3:** Summary of the combinations of PLA

	Modes of fabrication	Relevant properties	Advantages	Disadvantages	Refs.
PLA/HA	3D printing Electrospinning Air spray/jet spinning	Biocompatibility Porosity Versatility	Bone in crystalline phase is made by HA Allow blending with the bone Increases the Ca^2+^ in the cell Allow the proliferation of osteoblasts and promote cell growth Can be used as carrier scaffolds to transport proteins	Poor cell adhesion of PLA at its own Discarded for cell growth	[176–181]
PLA/PGA	Biografts Electrospinning	Biocompatibility Versatility Biodegradation rate depends on molecular weight	PLA/PGA is an excellent material for cell growth Allows osteoblast regeneration PLA/PGA mixture is convenient for cell adhesion Topology modifications enhance cell proliferation, adhesion, and differentiation	Fast degradation rate Risk of inflammation The process of crosslinking in hydrogels sometimes is not effective	[118, 182–185]
PLA/PBAT	Electrospinning Biomembranes	Tensile strength Versatility Biocompatibility Accessible mechanical properties Porosity Water permeability Interconnected microporous	Wide range of applications Favor the proliferation rate	The mean diameter of the PLA/PBAT scaffolds decreased while the mechanical properties improved	[129, 186, 187]
PLA/PEG	Micelles 3D printing Electrospinning	Hydrophilicity Ductility Flexibility Porosity Versatility Biodegradation by hydrolysis Can repel protein	PEG improves the biocompatibility of the copolymers with which it is mixed Help for cell proliferation and oxygenation PEG has good miscibility with organic solvents	Poor cell adhesion	[185, 188–192]
PLA/Lignin	Electrospinning Nanoparticles	Antimicrobial Antioxidant Anti-ultra-violet (UV) Biocompatibility Non-toxicity Porosity	Enhance the mechanical properties of the copolymers	The use of high concentrations of sodium chloride used as a solvent cause phase separation	[143, 190, 193–196]
PLA/PPy	Electrospinning Hydrogels	Conductivity Biocompatibility Biodegradation	Electrically stimulate the proliferation, adhesion, and cell growth in potential electroactive tissues	Low solubility The PPy tends to be fragile	[192, 197, 198]
PLA/Chi	Electrospinning Biomembranes Micelles Hydrogels Nanoparticles	Biocompatible Biodegradable Antibacterial activity Porosity Gel-forming properties High affinity with macromolecules	It is a natural polymer made from renewable sources Allows wound healing	It has poor cell recognition sites and low hydrophilicity Poor mechanical properties	[162, 196, 199–201]
PLA/PCL	Electrospinning Biomembranes Inkjet technology 3D printing	High purity Adequate processing Excellent mechanical properties Biocompatibility Biodegradation	The expression levels of elastin, angiopoietin, laminin-4α and − 5α increased in PCL and PLA nanofibers without any exogenous factor	Degradation products can be reabsorbed PLA/PCL are less hydrophobic and have less resistance to traction	[168, 199, 202–204]

### The emphasis on recent advances in PLA derivatives

Recently, several works have highlighted the most innovative developments for PLA derivatives. Ren et al. [205] reported a bimodal cell structure PLA/ cellulose nanocomposite synthesized by depressurization foaming as a possible thermal insulation prospect material. Another significant innovation is the production of chitosan/collagen hydrogel scaffolds from 3D printed PLA strut and cellulose nano-fibers intended for use in cartilage tissue engineering. The composite showed no cytotoxic effect on mesenchymal stem cells and enabled cell growth, attachment, proliferation, and migration through the scaffolds [206]. Also noted is the use of a freeze-drying technique to prepare a PCL/PLA scaffold containing zirconium (n-ZrO_2_) nanoparticles. The scaffolds were subsequently coated with polypyrrole and then enhanced their hydrophilicity and supported in vitro human corneal epithelial cell viability, attachment, and proliferation, suggesting a possible use in regenerative medicine [207]. Lastly, Ye et al. called attention to the PCL/ PLA/ microcrystalline cellulose composites fabricated via extrusion technology. The innovative development exhibited high biocompatibility and adhesion of human breast cancer cells, indicating a bright future in bioengineering research [208].

On the other hand, some current research themes regarding PLA-based materials are the shape memory effect, the piezoelectric properties, and the injectability. The shape memory effect is the polymer’s ability to change from the initial shape to a stress-free form. The latter state maintains the shape recovery until it is triggered externally with a stimulus. 4D printing implies changing the functionality or structural property of PL-based biomaterial tridimensionally printed [209]. Polybutylene succinate/PLA composite filament was prepared by 4D printing, and the scaffold showed potential for use in tissue engineering [210]. Maleic anhydride grafted onto PLA was used as a compatibilizer (2 wt%) on the shape memory abilities of poly(ethylene glycol)/ PLA blends, allowing for the biomaterials to be optimized for usability as scaffolds with improved chain entanglement and interfacial adhesion [211].

In recent years, researchers have paid much attention to the electroconductive and piezoelectric properties and behavior of PLA-based materials for their potential to mimic bone tissues. Ferroelectrets films created from PLA can be used to prepare biosensors involved in the development and growth of cells [212]. A biphasic layered structure was synthesized from electrospun mats of piezoelectric polyvinylidene fluoride/PLA embedded in a terpolymer hydrogel of alginate/gelatin/polypyrrole-grafted-gelatin to form osteon-mimetic samples [213]. Biopiezoelectric materials, such as barium titanate/ PLA composites, mimicked the microenvironment of bone tissue successfully and guided it through regeneration [214]. Concerning the injectability of PLA-based polymers, the scientific community has found that these biomaterials can act as an aqueous reservoir to treat defect shapes in bone tissue engineering because they can be molded easily and exhibits good biodegradability, rheology, selectivity, and targeting capability [212, 215–217].

**Fig. 8:**
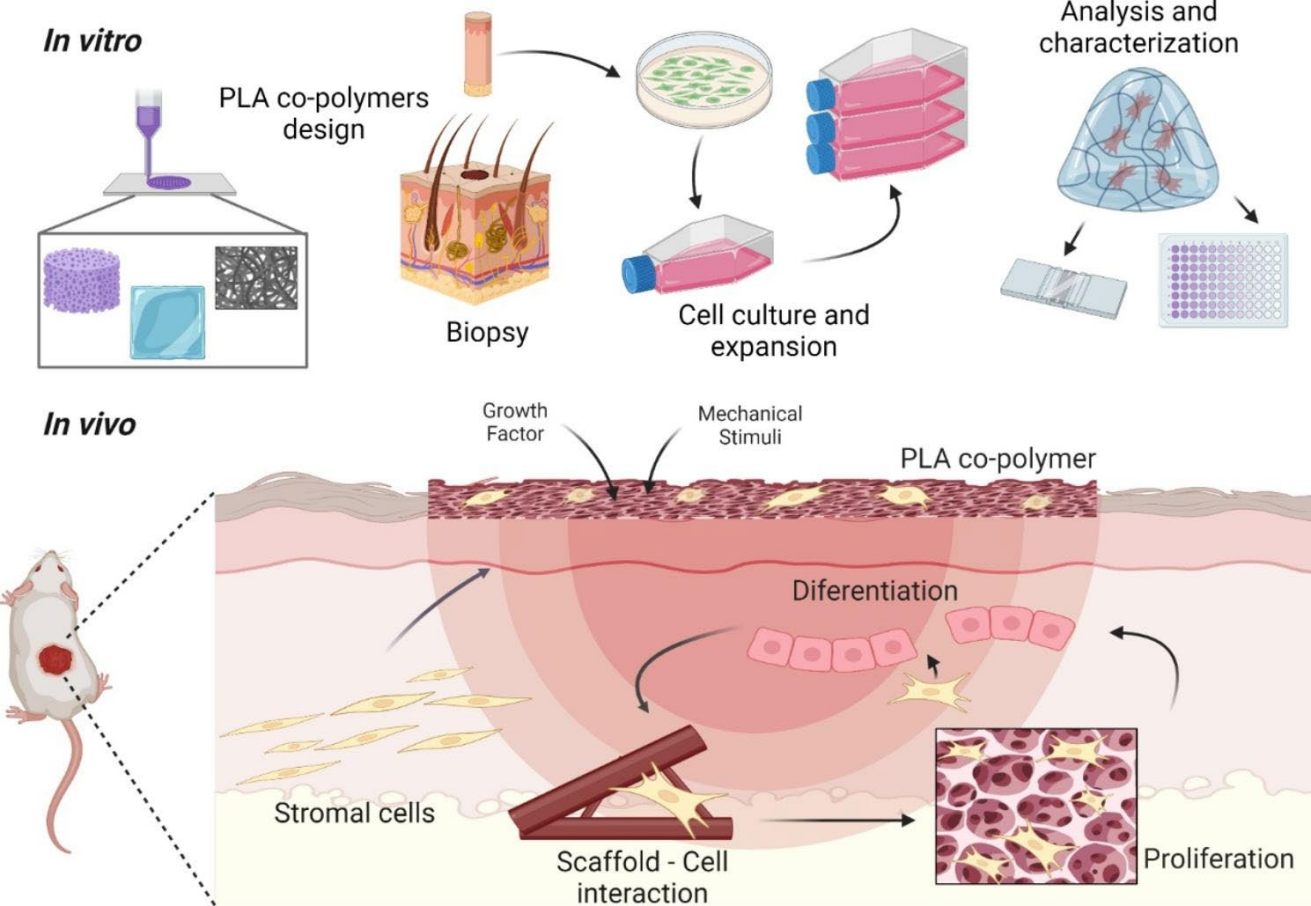
PLA and PLA-based materials designed for in vitro and in vivo performance

Figure 8 represents the performance of PLA-based materials added with antimicrobials for in vitro and in vivo research. A skin biopsy provides autologous dermal fibroblast that can be combined with PLA copolymers after cell culture and expansion to produce wound dressings. The design of new types of PLA derivatives architecture supplies the necessary micro-environment for wound healing [218, 219].

Some of the most recent and exciting works found in the literature are the in vitro increase of the bioactivity and interface strength of PLA hybrid coatings which was reinforced with hydroxyapatite – Al_2_O_3_ [220]; the inhibitory capacity against S. aureus. and E. coli, the antioxidant capacity, and the interphase compatibility were improved in vitro for novel poly(butylene succinate)/ PLA blend combined with glycyrrhetinic and rosmarinic acids as compared to neat PLA [221]; and the in vitro and in vivo study of the antiocratoxigenic and antifungal efficacy of PLA fibbers containing Ocimum gratissimum L. and Ocimum basilicum L. against Aspergillus niger and Aspergillus carbonarius [222].

## Limitations of the PLA as a biomaterial

PLA is a versatile polymer with many applications in various areas. Despite this, working with PLA can have disadvantages that must be considered since being a semi-synthetic polymer; it can have problems such as the lack of recognition of cell signaling, lack of adhesion, or even hydrophobicity that affects tissue development in vivo depending on the applications in which it is involved. Not to mention that the same advantage provided by the degradability of PLA can be a disadvantage since, being volatile and prone to hydrolysis, it may not be viable for specific biomedical applications [223, 224].

Moreover, it is expensive compared to petrochemicals, with a more intense manufacturing method and a lower yield than conventional polymers. The PLA working temperature is pretty low, and the co-blending is challenging to implement.

PLA is known for its controllable degradation rate and non-toxic components of degradative products. However, PLA-based degradation behavior depends on the molecular weight and glass transition temperature. Therefore, a feasible procedure must be attained to avoid undesirable degradation products towards in vitro and i*n vivo* performance. Acid degradation by-products may produce inflammatory reactions and low cell affinity. It is thus crucial to prepare new added-value PLA biomaterials considering these issues.

## Conclusion

The capability of the human being to regenerate damaged tissues by itself is limited. Today diseases are fought and studied differently. Therefore, it becomes more complex to determine an effective treatment that is effective, efficient, and highly accurate. In addition, treatments must be friendly to the environment and low cost. Thus, PLA is an attractive biopolymer due to its multiple properties (biocompatibility, biodegradation, mechanical strength, non-toxicity) that benefit when developing new treatments within tissue engineering. It should be noted that these properties can be enhanced according to the modifications that can be made to PLA when mixed with other polymers, which opens up a broad spectrum of possibilities for the potential treatment of diseases.

Conclusively, the current trends in PLA-based research that are influencing the future of tissue engineering applications are the evolution of conventional electrospinning to needleless electrospinning, such as ultrasound-enhanced electrospinning, edge electrospinning (jet spinning), and near-field electrospinning to produce high-quality nanofibers scale-up; the scope widening by the synthesis of new tailored devices in the form of nano- and micro- capsules, particles, and hydrogels; the preparation of cutting-edge membranes for skin tissue engineering; the advances in 3D and 4D printing technologies; and the versatility in forming new types of complex composites and scaffoldings. This review is a good roadmap for implementing new approaches to strengthen the biomedical fields through the invention of feasible medical surgeries, derma, membranes covering, cosmetics, tissue engineering, and scaffolding. It is also worth mentioning that PLA represents a carbon emissions reduction, another point in its favor, while multiple research opportunities are waiting to be discovered.

## Data Availability

Not applicable.
